# Book Review: Legumes under Environmental Stress: Yield, Improvement and Adaptations

**DOI:** 10.3389/fpls.2016.00798

**Published:** 2016-06-03

**Authors:** Naser A. Anjum

**Affiliations:** Department of Chemistry, CESAM-Centre for Environmental and Marine Studies, University of AveiroAveiro, Portugal

**Keywords:** environmental stress, legumes, stress tolerance, yield improvements, molecular biology

Proteins are among the major constituents of a healthy and balanced human diet. Legumes have been serving as a human food-source for many centuries (Imsande, 2003). Notably, grain legumes (pulses) are very rich in protein-types and contribute to the human protein requirements as a primary and affordable source of proteins and minerals (Bohra et al., 2014). The United Nations has declared 2016 as the International Year of Pulses and also advocated pulses as nutritious seeds for a sustainable future (FAO, 2016). However, varied stress factors can hamper the legume-productivity because many of the world's most important food legumes are grown in arid and semi-arid regions of the world (Varshney et al., 2009). Taking aforesaid points into account, entitled above edited book aimed to: (a) discuss environmental stress responses and underlying mechanisms of major legumes; and (b) highlight important avenues for legume health-improvement and stress-adaptation.

Though not done by the editors chapters discussed herein can be divided into two major groups. Comprising nine contributions, the first group gives insights into the legume stress responses. The second group, with eight contributions, highlights approaches for improving legume stress tolerance.

Major insights into legume responses to a number of abiotic stresses including salinity, nutrient-deficiencies, pesticides, and temperature regimes are nicely overviewed. The impact of increasing soil salinity on legume crop health and productivity is particularly important, due to the sensitivity of legumes to salinity stress. Information related to leguminous crop genome sequences has also been well-summarized. The responses of legumes to nutrient deficiencies are elaborated, including their control measures with major micro and macro mineral nutrients. Osmotic stress is argued to be a factor impacting the legume–rhizobia symbiotic relationship by severely impairing nodule integrity and functioning. Two chapters are dedicated to discussion of the responses of chickpea (the third most important pulse crop) to major abiotic and biotic stresses. An overview is presented of chickpea responses to temperature regimes. The lack of simple and accurate screening procedures for screening parental genotypes and breeding populations for various abiotic stresses is advocated as a major bottleneck in the development of low temperature stress-tolerant chickpea. Contributions also provide details on the pesticide uptake, metabolism, persistence, and impacts in major leguminous crops. It is revealed that pesticides can severely impair biological nitrogen-fixation by impacting nodule formation and nitrogenase activity, causing oxidative stress, and damaging important components of the antioxidant defense system. Exploration of pesticide-tolerant bacterial strains is emphasized to improve legume–rhizobia association, and also to increase growth and performance of legume plants under pesticide stress.

Under the second group of contributions, several chapters highlight the role of exogenous application of phytoprotectants (including abscisic acid, and the approach of employing molecular/genetic protocols) in the minimization of abiotic stress impacts on legumes. In particular, the use of biotechnological approaches is argued as a panacea for overcoming biotic and abiotic stress constraints in legumes. The need for development of safer and more effective methodologies for legume stress tolerance improvement is emphasized. The isolation of a sufficient number of stress resistance genes, understanding their accurate delivery and analyses methods, and comparative analyzing protein expression profiles and functional analyses are argued to be significant in this context. Molecular-genetic information is meager on the legume biology and also with nodule formation, and other vital processes like resistance, potential tolerance, and susceptibility to various stresses (Langridge and Fleury, 2011). Hence, the chapters argue that the “omics” approaches comprising genomics, transcriptomics, proteomics, genomics, transgenomics, and phenomics can make transgenic breeding and genetic modification of legumes possible. Reports available on the above aspects are also very well-elaborated giving examples of the major legume crops.

Literature is extensive on the involvement of microRNAs (miRNAs) in the regulation of a wide variety of physiological and stress responses in non-legume crops (Zhang, 2015). However, miRNA has not been extensively researched in legumes. The role of miR166, miR169, and conserved miRNAs such as miR156, miR160, miR167, miR172, miR398, and miR399 in the infection, symbioses, and nodule development is critically discussed in some chapters. It is also highlighted that the studies on miRNAs in legumes can greatly help us to understand mechanisms of legume responses and tolerance to major stresses such as soil-salinity, drought, extreme temperature, metals/metalloids, and nutrient deficiency. Contributions advocate adopting new approaches (such as marker-assisted selection and marker-assisted gene pyramiding) in future research to develop legume genotypes having desirable genes, and with enhanced tolerance to numerous fungal and viral diseases, and extreme environmental conditions. Using several examples it is discussed how the worldwide increasing P-deficiency of alkaline calcareous soils can be combatted by adsorption isotherm techniques for legume crops in arid environments. Microbial strategies are also emphasized to be considered for the improvement of legume production in hostile environments including water deficit and soil salinity. Additionally, to improve yield and nodulation under hostile environmental conditions, deliberations advocate employing the strategy of co-inoculating the rhizobia-inoculated leguminous crops with plant growth-promoting rhizobacteria.

Notably, under field conditions, a number of biotic stresses can co-occur and modulate abiotic stress-impacts on plants, where interaction between these stress-types can yield positive (cross-tolerance) or negative outcomes (Atkinson and Urwin, 2012). However, less emphasis is given herein on the discussion highlighting the outcomes of the potential interaction of biotic and abiotic stresses, and their relationship with legume crop health and productivity. Unfortunately, most of the chapters are not focused and have failed to introduce their topics and highlight the state-of-the-art available on the topics discussed therein. Also, recent literature available on the subject has not been included in most chapters.

Since a thorough understanding of plant stress responses and tolerance is essential, new dialogue will be provoked by this book leading to improved legume health under adverse conditions. In summary, this book is timely and a welcome addition to the literature. Researchers and Ph.D. students in the field of crop-biology/physiology, agronomy, and plant-breeding areas can be benefited with this book.

This book can be found at: http://onlinelibrary.wiley.com/book/10.1002/9781118917091.

## Author contributions

NAA conceived the idea and wrote the paper.

### Conflict of interest statement

The author declares that the research was conducted in the absence of any commercial or financial relationships that could be construed as a potential conflict of interest.
